# Per-Pixel Forest Attribute Mapping and Error Estimation: The Google Earth Engine and R *dataDriven* Tool

**DOI:** 10.3390/s24123947

**Published:** 2024-06-18

**Authors:** Saverio Francini, Agnese Marcelli, Gherardo Chirici, Rosa Maria Di Biase, Lorenzo Fattorini, Piermaria Corona

**Affiliations:** 1 Department of Agriculture, Food, Environment and Forestry, University of Firenze, 50145 Firenze, Italy; gherardo.chirici@unifi.it; 2 NBFC, National Biodiversity Future Center, 90133 Palermo, Italy; rosa.dibiase@unisi.it; 3 Department for Innovation in Biological, Agro-Food, and Forest System, Tuscia University, 01100 Viterbo, Italy; agnese.marcelli@unitus.it; 4 Fondazione per il Futuro delle Città, 50127 Firenze, Italy; 5 Department of Economics and Statistics, University of Siena, 53100 Siena, Italy; lorenzo.fattorini@unisi.it; 6 CREA Research Centre for Forestry and Wood, 52100 Arezzo, Italy; piermaria.corona@crea.gov.it

**Keywords:** remote sensing, Google Earth Engine, R software, forest mapping, bootstrap

## Abstract

Remote sensing products are typically assessed using a single accuracy estimate for the entire map, despite significant variations in accuracy across different map areas or classes. Estimating per-pixel uncertainty is a major challenge for enhancing the usability and potential of remote sensing products. This paper introduces the *dataDriven* open access tool, a novel statistical design-based approach that specifically addresses this issue by estimating per-pixel uncertainty through a bootstrap resampling procedure. Leveraging Sentinel-2 remote sensing data as auxiliary information, the capabilities of the Google Earth Engine cloud computing platform, and the R programming language, *dataDriven* can be applied in any world region and variables of interest. In this study, the *dataDriven* tool was tested in the Rincine forest estate study area—eastern Tuscany, Italy—focusing on volume density as the variable of interest. The average volume density was 0.042, corresponding to 420 m^3^ per hectare. The estimated pixel errors ranged between 93 m^3^ and 979 m^3^ per hectare and were 285 m^3^ per hectare on average. The ability to produce error estimates for each pixel in the map is a novel aspect in the context of the current advances in remote sensing and forest monitoring and assessment. It constitutes a significant support in forest management applications and also a powerful communication tool since it informs users about areas where map estimates are unreliable, at the same time highlighting the areas where the information provided via the map is more trustworthy. In light of this, the *dataDriven* tool aims to support researchers and practitioners in the spatially exhaustive use of remote sensing-derived products and map validation.

## 1. Introduction

Accurate information on forest ecosystems is crucial for conservation, climate change mitigation, resource management, land use planning, understanding ecosystem services, and advancing research and education [1,2]. In particular, up-to-date and spatially detailed forest data are essential to address current global challenges [3,4] such as, e.g., deforestation and forest degradation [5].

The value of remote sensing in Earth ecosystem monitoring has been a topic of historical discussion as to whether it should be considered simply a “toy” or a powerful tool [6]. However, remote sensing has now become a widely adopted technology in many sectors, including forestry. For example, there are various readily available global forest cover and change products [7], as well as forest disturbance products covering large areas and periods spanning several decades [8,9,10,11,12]. These products are used in some jurisdictions to enhance estimates of official national monitoring programs, such as national forest inventories, and they provide spatially explicit assessments of forest attributes [13] and forest disturbances [14].

Integrating remote sensing and ground data for mapping forest attributes can be performed using two main approaches: model-based inference and design-based inference. The differences between these approaches are well discussed in the statistical literature (e.g., [15]). Design-based approaches are appealing for their objectivity, based on the fact that the sampling design and the associated probability distribution are not modeled but are well defined a priori [16]. However, purely design-based methods cannot be used to produce wall-to-wall maps at the pixel level due to their inability to make estimations with non-sampled pixels without making any assumption and without exploiting auxiliary variables. In fact, either a pixel is sampled, and then there is no need for estimation, or the pixel is not sampled, and we have no information to perform estimation [17]. This lack of information makes the use of design-based inference to produce per-pixel maps impossible. Consequently, model-based interpolation methods such as kriging predictors (including cokriging and regression kriging) and nonparametric techniques like locally weighted regression and k-nearest neighbor methods are usually used for mapping [16,18,19,20].

On the other hand, the use of models is not limited to model-based mapping alone. Indeed, following the model-assisted perspective, an assisting model can be exploited within a design-based inference [21]. In this context, a model is used to perform interpolation at unsampled pixels, but the uncertainty associated with the interpolated values still arises from the probabilistic sampling scheme used to select the pixels. Recent works have, in fact, moved in this direction, and first attempts have proposed to map continuous and finite populations of spatial units in a design-based framework [17,22]. Such methods exploit Tobler’s first law of geography [23] as an assisting model. As the model supposes that nearby units tend to be more similar than distant units, then the inverse distance weighting interpolator is used, providing more importance to neighboring locations.

The open accessibility of data and cloud computing platforms that enable the calculation of sophisticated information globally [24,25,26] characterize the current era of remote sensing. To improve the reliability of remote sensing products and exploit them in order to provide statistically rigorous estimates for large-scale or country reporting, huge efforts have been developed in recent years [27,28,29,30]. However, several limitations persist, leading many researchers and authorities to lean towards ground data acquisition. This is because, although map products derived from remotely sensed data are increasingly accurate and easy to provide, they may lack reliable quality indicators. Accordingly, Ref. [31] recently argued that clearer and more transparent map validation remains a major challenge in remote sensing for forestry. More specifically, remote sensing products are usually delivered with a single accuracy estimation for the whole map, while the accuracy can change greatly, depending on map areas.

In this context, this study aimed at matching ground and remote sensing data to produce maps of variables of interest and per-pixel estimates of the associated error. To this purpose, we introduce *dataDriven*, a Google Earth Engine [24], and R [32] tools for mapping forest attributes through a complete data-driven approach that exploits both Sentinel-2 remote sensing data as auxiliary information and ground data to produce both the attribute map at pixel level and the corresponding map of uncertainty estimates.

This paper is organized as follows. First, we introduce the new *dataDriven* tool (Section 2) by providing details on the input data and the allowed sampling designs to collect reference data (Section 2.1) and providing methodological details on how remote sensing data are used to produce variables that will serve as auxiliary information (Section 2.2). Second, we provide a schematic overview of how *dataDriven* combines reference and remote sensing data to produce per-pixel maps of the attribute of interest and the associated uncertainty (Section 2.3). Then, *dataDriven* is tested over a study area in Tuscany, Italy (Section 3). Remarks and future perspectives are presented in Section 4.

## 2. The *dataDriven* Open Source Tool

*dataDriven* is a Google Earth Engine [24] and R [32] tool for mapping forest attributes within a given area of interest (AOI) partitioned into pixels, some of which are visited in the field to measure/assess the attribute of interest (https://github.com/saveriofrancini/dataDriven, accessed on 11 June 2024). It is worth noting that, henceforth, the term pixel does not necessarily denote a satellite pixel, i.e., a pixel acquired via a satellite mission and with a specific size, but possibly an agglomerate of neighboring satellite pixels of suitable sizes to be exhaustively visited on the ground.

**Figure 1:**
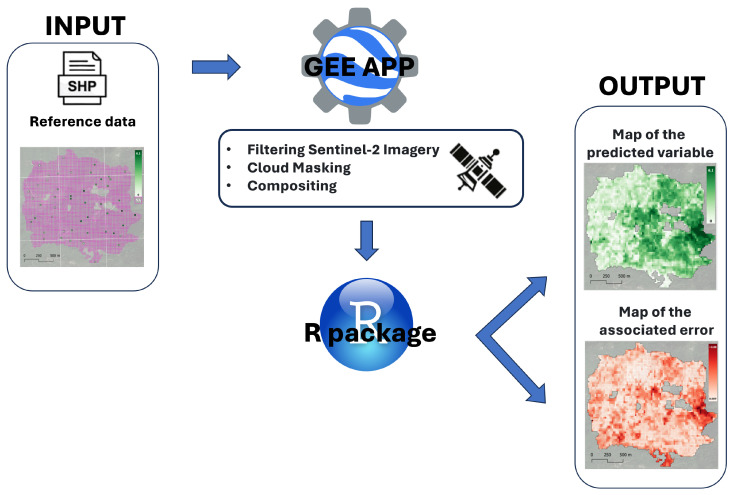
*dataDriven* workflow.

The *dataDriven* tool requires a specific sample reference dataset as an input (Section 2.1) and implements two main steps, for which specific details are provided in the following sections, while a summary is provided below, and a schematic representation is presented in Figure 1.

The first step (Section 2.2) involves the calculation of Sentinel-2 predictors to be used as auxiliary information, and it is implemented in the form of a Google Earth Engine (GEE) application. The GEE platform is a cloud-based solution that combines a huge, multi-petabyte collection of satellite images and geospatial datasets with the capability of large-scale planetary analysis [24]. Its global applications span various sectors, including forestry and forest disturbance monitoring [7,12,30]. GEE boasts three key advantages over other cloud-computing platforms. First, it offers flexibility by allowing users to bring their algorithms to data using high-level programming languages and high-performance computing. Second, it prioritizes scientific reproducibility due to expandable storage and processing capabilities. Third, its processing performance can be improved by incorporating additional resources without requiring users to modify their methods or code.

The second step of *dataDriven* (Section 2.3) is implemented in R [32] and delivered in the form of an R package available on GitHub. This step implements the data-driven, per-pixel mapping of both the attribute of interest and the associated uncertainty.

### 2.1. Input Reference Data

Our tool requires an input shapefile with ground data collected in the field for a sample of pixels selected following one of the next probabilistic sampling schemes:

- Simple Random Sampling Without Replacement (SRSWoR): sample pixels are randomly selected without replacement;
- One per Stratum Stratified Sampling (OPSS): the population is divided into blocks of contiguous pixels, and a pixel is randomly selected in each block;
- Systematic Sampling (SYS): the population is divided into equally shaped blocks containing the same number of pixels; then, a pixel is randomly selected in one block and repeated in the others.

For each pixel in the population, the input shapefile must include the following two attributes: (A) the ID of the block to which each pixel belongs and (B) the attribute of interest measured/assessed in the field within the sampled pixel. For non-sampled pixels, i.e., pixels for which the attribute of interest was not measured/assessed in the field, the label NA (not available) must be assigned to the attribute (B). In the case of SRSWoR, the attribute (A) must be a unique value for each pixel, as all pixels belong to a unique block coinciding with the whole population. Based on attribute (A), *dataDriven* automatically identifies which of the three sampling strategies has been used.

Among the three schemes, OPSS has historically been widely adopted [33] and is currently implemented in several national forest inventories (e.g., Italy and the USA), as it allows the sample pixels to be spread over the entire AOI. In fact, while SRSWoR can lead to over/under-representation of the AOI, OPSS reduces the probability of selecting neighboring pixels, ensuring spatially balanced samples with related advantages [34].

### 2.2. Sentinel-2 Data and Derived Predictors

The Sentinel-2A and 2B satellites together offer a frequency of five days—up to 2–3 days at mid-latitudes—and multispectral bands with a spatial resolution ranging from 10 to 60 m. The GEE *dataDriven* application (https://code.earthengine.google.com/?accept_repo=users/saveriofrancini/PRIN, accessed on 11 June 2024) exploits visible (red, green, and blue) and near-infrared (nir) bands—with ten-meter resolution—and red edge (redE1, redE2, redE3, and redE4) and short-wave infrared (swir1 and swir2) bands—with 20-meter resolution.

*dataDriven* preprocesses Sentinel-2 data to construct cloud-free composites over different periods. The main steps involve (1) the filtering of Sentinel-2 imagery, (2) cloud masking, and (3) creating pixel-based composites [30]. *dataDriven* can filter Sentinel-2 imagery by specifying the period, the years of interest, and the maximum percentage of clouds. Then, clouds are masked out using the Sentinel-2 cloud probability dataset [35], and cloud-free composites are generated for the selected years as the medoid of all remaining valid observations. The medoid algorithm populates the image composite with the satellite pixel with the surface reflectance most similar to the median surface reflectance value for that pixel [8]. For details on compositing parameters and resulting composite assessments, see [36]. As a result, medoid predictors are calculated for the selected periods and years and each pixel in the input shapefile (Section 2.1). Since Sentinel-2 satellite pixels are ten meters in size, and since each pixel can include several satellite pixels, depending on the sample size, the medoid predictors are calculated for each pixel as the average of all the satellite pixels included. Furthermore, the latitude and longitude of each pixel are downloaded from GEE and included among the shapefile predictors, as they are subsequently necessary to implement the inverse distance-weighting interpolation (see Section 2.3).

### 2.3. Per-Pixel Mapping and Associated Error Mapping

Statistical details, equations, and mathematical proofs of the procedure adopted to perform per-pixel mapping are described in [37], while here, we provide a schematic and technical overview of the main implemented steps.

1. Exploiting the Akaike-type criterion [38], a variable selection process is implemented to remove Sentinel-2 auxiliary variables that are poorly correlated with the attribute of interest or that are strongly correlated with each other, thus providing little additional information.
2. Using the selected variables, a regression model is adopted to estimate the coefficients in order to predict the variable of interest as a linear combination of the auxiliary variables for each pixel in the population.
3. For the sampled pixels, residuals are computed as the difference between the regression predictions and the true attribute recorded on the ground. Residuals for non-sampled pixels are estimated using the inverse distance weighting interpolator. For each pixel in the population, residuals are then added to the predicted values to achieve the map of the forest attribute.
4. The Horvitz–Thompson total estimate and the total estimate achieved from the map by summing the interpolated values are calculated. Then, the map is harmonized based on the ratio of the two estimates so that the sum of the pixel estimates in the map coincides with the Horvitz–Thompson estimate [39]. This is the *final map* of the interest attribute produced via *dataDriven*.
5. To produce the error map, a minimum of 1000 independent samples are selected via bootstrap resampling from the *final map*. Each sample has the same size as the initial sample for which reference data were available and is selected following the same sampling scheme (Section 2.1). If, for example, the initial sample is made using 50 pixels selected via OPSS, each new bootstrap sample will include 50 pixels selected via OPSS. A new map of the attribute of interest is then obtained for each bootstrap sample by implementing steps 1 to 4. The uncertainty map is finally produced by calculating the root mean square error from the bootstrap maps for each pixel.

## 3. *dataDriven* Implementation: A Case Study

The *dataDriven* tool was tested on a forested AOI (the Rincine forest estate) located in eastern Tuscany in Central Italy, centered on coordinates 43.9 N and 11.6 E. *dataDriven* was used to predict the forest volume density and estimate the root mean square error for each pixel, but we stress that it can be applied to any kind of variable of interest or study area. In this study, we used the reference dataset adopted in [37].

To select the sample to be visited in the field, the AOI was tessellated using a grid of 5449 square pixels of 23 m × 23 m. Sentinel-2 pixels were resampled from 10 m to 23 m using bilinear interpolation [40]. OPSS was subsequently implemented. Details on the OPSS procedure are shown in Section 2.1, while in the following, details are given about the case-study implementation.

**Figure 2:**
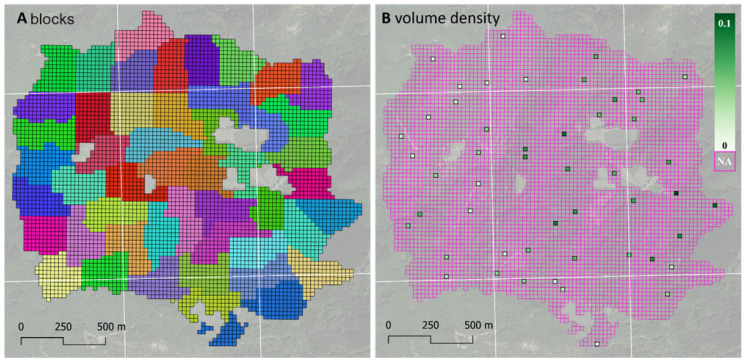
Input shapefile. Panel (**A**) shows the blocks, with each of the 50 blocks shown with a different color. Panel (**B**) shows the wood volume density (m^3^/m^2^) measured in the field at each sampled pixel.

Following OPSS, the population of 5449 pixels was divided into 50 blocks: 49 blocks containing 109 adjacent pixels each and one block containing 108 pixels (Figure 2, left). Of the 5449 pixels, 340 were located in non-forest areas and excluded from the population; the remaining population eligible for sampling was composed of 5109 pixels distributed across blocks of varying sizes: nine of the 50 blocks contained fewer than 100 pixels, with the smallest block comprising 57 pixels. Then, a sample pixel was randomly selected for each block.

Field measurements were conducted within the sampled pixels over the period from June to November 2016. Species identification, stem diameter at breast height (DBH), and stem height were assessed for each tree with a DBH greater than 2.5 cm. The wood volume of each tree was estimated using allometric models derived from the Italian National Forest Inventory [41]. Following typical forest survey practices, the uncertainty associated with tree volume predictions was disregarded, and the predicted volumes were treated as fixed values [18,42]. For the sampled pixels, density was reported and used as the dependent variable, calculated as the ratio between the total wood volume recorded within a pixel and the pixel size (i.e., 23 m × 23 m or 529 m^2^). From this ground survey, we obtained the input reference dataset required for *dataDriven* as an input (Figure 2).

**Figure 3:**
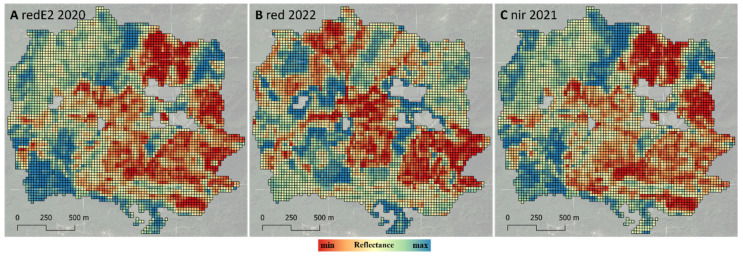
Example of three of the 30 Sentinel-2 predictors downloaded from GEE. Each of the panels (**A**–**C**) shows a different predictor.

The Sentinel-2 predictors were calculated via the GEE *dataDriven* application. The GEE *dataDriven* app permits the setting of several parameters to construct the medoid composite and then calculate the predictors. In this study, default parameters were used to test the application. For each year between 2020 and 2022—Start/End years parameters in the GEE user interface—we selected all images acquired between June 10 and August 20—Start/End date for composite—and with cloud cover of less than 20%—the Clouds threshold parameter. Then, by clicking on the run button, a medoid composite was automatically calculated for each year (Section 2.2), and a new shapefile including 30 Sentinel-2 predictors was downloaded (Figure 3). There were 30 predictors since there were ten Sentinel-2 bands (Section 2.2) and three medoid composites (2020 to 2022).

**Figure 4:**
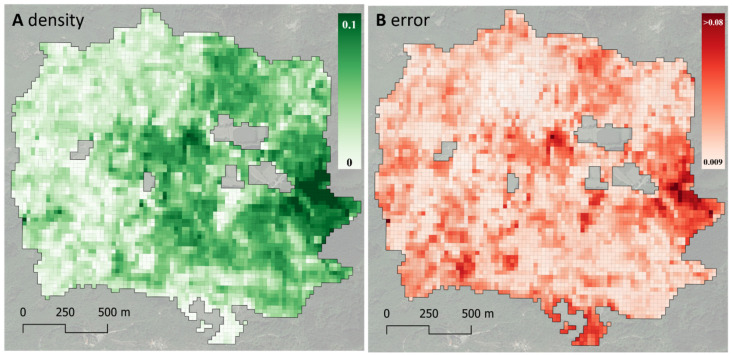
Panel (**A**) shows the predicted wood volume density map. Panel (**B**) shows the associated predicted per-pixel root mean square error.

Finally, the *dataDriven* R package was used, which implements the procedure described in Section 2.3 and produces the map of the attribute of interest and the map of the associated error (Figure 4). The average volume density was 0.042, corresponding to 420 m^3^ per hectare, and it ranged between 0 and 1455 m^3^ per hectare. *dataDriven* estimated an average per-pixel error of 0.0285 in terms of density, or 285 m^3^ per hectare. The estimated pixel errors ranged between 93 and 979 m^3^ per hectare.

## 4. Future Perspectives

This paper has introduced *dataDriven* (https://github.com/saveriofrancini/data/Driven, accessed on 11 June 2024), an open-access tool for producing per-pixel maps of the interest attribute, as well as per-pixel estimates of their root mean squared errors (e.g., Figure 4A,B). Several products providing information on pixel quality exist. Most of them only concern atmospheric noise, giving information on per-pixel quality, as for example, the bands of satellites like Sentinel-2 and Landsat [43,44,45]. Other products concern the per-pixel accuracy of maps that, as usual in forest studies, are obtained using geostatistical or machine learning approaches. In the case of geostatistical mapping, accuracy estimation is performed from a series of more or less realistic model assumptions (e.g., [15], Equation (20.15), while in the case of machine learning procedures, accuracy estimation is usually performed from empirical cross-validation procedures [30] but lacks theoretical foundations. On the other hand, the procedure we adopted in this study stems from a theoretical study by [46] and provides per-pixel, statistically rigorous, design-based estimates of map accuracy.

*dataDriven* combines the planetary-scale computing capabilities of GEE with the R programming language and integrates the ground-measured data of the attribute of interest with Sentinel-2 data used as auxiliary variables to produce the per-pixel map of the attribute of interest and associated error for the entire AOI.

The ability to produce error estimates for each pixel in the map is a novel aspect provided via *dataDriven* in the context of the current advances in environmental monitoring and assessment: per-pixel uncertainty informs users about areas where the map estimates are unreliable, at the same time highlighting the areas where the information provided via the map is trustworthy; therefore, it constitutes support not only from an analytical point of view but also as a powerful communication tool. On the other hand, in this study, we obtained an average error of 285 m^3^ per hectare, indicating that further research is necessary to enhance map accuracies. Future efforts could focus on incorporating additional predictors from other available sensors [47] or exploiting data collected over longer time series.

*dataDriven* implements a statistically sound procedure of a design-based nature [37] following the model-assisted perspective such that the resulting precision is objective, as it is determined according to the sampling scheme actually adopted to select pixels for the ground measurement of the attribute of interest, without any assumption.

Based on open-access Sentinel-2 data, *dataDriven* can be applied anywhere in the world as long as input-reference (ground-measured) data are available. More remotely sensed variables can be easily added, calculated, and downloaded from the GEE side, based on several other satellite missions, and possibly also exploiting non-optical data, thus making the most of the huge catalog of open access data available in GEE.

Ground-measured reference data can be collected through three different sampling schemes, i.e., tessellated schemes (SYS and OPSS) that are the most common in forest surveys (Section 2.1). While additional sampling schemes may be added to *dataDriven* in the future, the sampling scheme must be specified: this can be an issue in the case of available reference data for which the sampling scheme is unknown or non-probabilistic.

The current version of *dataDriven* exploits simple regression models to predict the attribute of interest for all pixels in the population. Still, future releases may be able to integrate further techniques, including machine learning and artificial intelligence imputation.

*dataDriven* takes advantage of parallel computing and allows us to make the most of the available computational capabilities. *dataDriven* outputs are generated very quickly, from a few minutes to a few hours, depending on the size of the population. In our case study (about 300 hectares), the predictors were downloaded from GEE in less than one minute, while the per-pixel maps of the wood volume density and that of the associated error (estimated using 1000 bootstrap resamplings) were obtained via the R package in a minute and ten seconds, using a standard workstation (12th Gen Intel Core i9-12900K) with 16 cores, 24 logical processes, and 30 GB of RAM.

## 5. Software Details

- Name of tool: *dataDriven*.
- Developers: Saverio Francini, Agnese Marcelli, and Rosa Maria Di Biase.
- Year first available: 2023.
- Hardware required: Basic computer.
- Requirements: The R software (https://www.r-project.org/, accessed on 11 June 2024), and a Google Earth Engine account (https://earthengine.google.com/, accessed on 11 June 2024).
- Source Code Availability: Codes are available on GitHub (https://github.com/saveriofrancini/dataDriven, accessed on 11 June 2024).
- Data availability: *dataDriven* analyzes (i) Sentinel-2 images that are open and freely available on Google Earth Engine (https://developers.google.com/earth-engine/datasets/catalog/COPERNICUS_S2_SR_HARMONIZED, accessed on 11 June 2024) and (ii) ground data—for which we provide a test dataset on GitHub (https://github.com/saveriofrancini/dataDriven/tree/master/inst/data, accessed on 11 June 2024).
- Cost: Free.
- Program languages: R and JavaScript.

## Data Availability

The original contributions presented in the study are included in the article. Further inquiries can be directed to the corresponding author.
